# Airway Clearance and Pediatric Pulmonary Rehabilitation in Primary Ciliary Dyskinesia: A Clinical Framework for Children and Adolescents

**DOI:** 10.3390/jcm15176676

**Published:** 2026-08-28

**Authors:** Jiahui Zhao, Lina Chen, Wenhao Yang, Hanmin Liu

**Affiliations:** 1 Department of Pediatric Pulmonology, West China Second University Hospital, Sichuan University, Chengdu 610041, China; jadezhao0920@gmail.com (J.Z.); chenlina_78@163.com (L.C.); 2 Key Laboratory of Birth Defects and Related Diseases of Women and Children, Sichuan University, Ministry of Education, Chengdu 610041, China; 3 NHC Key Laboratory of Chronobiology, Sichuan University, Chengdu 610041, China; 4 Department of Pediatric Pulmonology and Immunology, WCSUH-Tianfu·Sichuan Provincial Children’s Hospital, Sichuan University, Meishan 620010, China; 5 The Joint Laboratory for Lung Development and Related Diseases of West China Second University Hospital, Sichuan University and School of Life Sciences of Fudan University, West China Institute of Women and Children’s Health, West China Second University Hospital, Sichuan University, Chengdu 610041, China; 6 Sichuan Birth Defects Clinical Research Center, West China Second University Hospital, Sichuan University, Chengdu 610041, China; 7 Xizang Region Child Development Clinical Medical Research Center, Lhasa 850000, China

**Keywords:** primary ciliary dyskinesia, airway clearance techniques, pediatric pulmonary rehabilitation, age-stratified care, exercise training, bronchiectasis, mucociliary clearance

## Abstract

Primary ciliary dyskinesia (PCD) is an inherited motile-cilia disorder. Impaired mucociliary transport promotes persistent secretion retention, infection, inflammation, and bronchiectasis. The central treatment problem is therefore failure of airway clearance rather than infection alone. This narrative review evaluates airway clearance techniques (ACTs), mucoactive adjuncts, and pulmonary rehabilitation for children and adolescents. We prioritize direct pediatric PCD evidence, identify mixed-age or adult PCD findings as population-bound PCD evidence, and label pediatric bronchiectasis and cystic fibrosis evidence as indirect. Because no ACT has been shown to be universally superior and pediatric rehabilitation evidence remains limited, we present an author-proposed clearance-anchored dual-pillar framework. ACTs directly address mucus retention. Pulmonary rehabilitation complements ACT by targeting fitness, muscle performance, participation, and self-management. Response should be assessed across four domains: clinical stability, treatment performance, physiological and functional outcomes, and patient and family experience. Prospective multicenter comparative trials and harmonized pediatric outcome measures are urgently needed.

## 1. Introduction

Primary ciliary dyskinesia (PCD) is a genetically heterogeneous disorder of motile cilia that impairs mucociliary transport. The respiratory manifestations of PCD usually appear early in life, including neonatal respiratory distress [1], persistent wet cough, nasal congestion, recurrent middle-ear disease, and progressive suppurative airway disease [2,3]. Accordingly, routine, individualized airway-clearance management should be established and reviewed regularly [4,5], particularly because structural lung disease may progress despite apparently stable spirometry [6].

The core treatment problem in PCD is persistent mucus-transport failure, not infection alone. Abnormal motile-cilia function impairs mucociliary transport in PCD, promoting secretion retention, recurrent infection, chronic airway inflammation, and progressive bronchiectasis [4,7]. Antibiotics are essential for pulmonary infections and exacerbations [4], but they neither restore ciliary transport nor mechanically remove retained secretions. Regular airway clearance is therefore a pathophysiologically anchored component of care [4,5]. PCD and cystic fibrosis (CF) share downstream manifestations, but their initiating defects differ. PCD involves motile-cilia dysfunction; CF involves CFTR-related ion and fluid transport abnormalities [7]. Evidence transferred from CF should therefore be explicitly identified as indirect [8].

Previous reviews and consensus documents have summarized PCD diagnosis, treatment, and multidisciplinary management or examined the transferability of CF airway-clearance evidence [5,8,9,10]. They do not, however, provide a developmentally stratified pulmonary rehabilitation framework across childhood and adolescence. The present review therefore addresses a narrower pediatric implementation question: how ACT and developmentally adapted pulmonary rehabilitation can be integrated as complementary, non-substitutable components of care.

Airway clearance techniques (ACTs) mobilize secretions toward central airways for removal by huffing, coughing, or expectoration. Generic ACT classifications include caregiver-assisted techniques, breathing-based expiratory-flow strategies, positive expiratory pressure (PEP), oscillatory PEP (OPEP), and external oscillatory devices [8,11]. However, a 2024 systematic review found very low-certainty evidence and no clear lung-function advantage of device-supported approaches over conventional chest physiotherapy [12]. Clinical decisions consequently depend on a combination of PCD-specific studies, expert consensus, guideline-derived recommendations, physiological rationale, and indirect evidence from pediatric bronchiectasis or CF.

Mechanical clearance does not address all consequences of chronic respiratory disease. Studies of PCD cohorts have documented reduced aerobic fitness, respiratory muscle performance, habitual activity, and functional capacity in some children and young people [13,14]. A small pediatric randomized trial also suggests that a structured activity modality can improve selected short-term functional outcomes [15]. Pulmonary rehabilitation is therefore considered here as a complementary functional intervention rather than a substitute for ACT: it targets exercise capacity, muscle performance, participation, behavioral support, and progressive self-management.

Direct pediatric PCD trials are few and often small and short. Mixed-age, adult, and indirect disease evidence answers narrower questions and cannot establish pediatric PCD efficacy.

The review adds an author-proposed pediatric framework with three features. First, it treats ACT and pulmonary rehabilitation as complementary, non-substitutable pillars. Second, it adapts implementation to developmental capacity and progressive self-management. Third, it links explicit evidence provenance to four-domain monitoring and reassessment. The framework organizes individualized care and research questions across childhood and transition. It is not a validated algorithm, consensus pathway, or guideline.

**Literature search and evidence selection:** This narrative review was informed by a structured, non-systematic search of PubMed/MEDLINE, Embase, and the Cochrane Library from inception to 24 June 2026. The search combined “primary ciliary dyskinesia” or “Kartagener syndrome” with broad topic terms. These included “child,” “adolescent,” “treatment,” “management,” “airway clearance,” “pulmonary rehabilitation,” “exercise,” and “monitoring.” Boolean operators were adapted to each database. Reference lists of relevant guidelines, consensus statements, reviews, and key studies were also examined. Titles, abstracts, and relevant full texts were assessed for relevance to pediatric PCD management, particularly airway clearance, pulmonary rehabilitation, monitoring, and emerging therapies. Clinical studies, reviews, guidelines, and consensus statements were considered. Direct pediatric PCD evidence was prioritized. When it was insufficient, mixed-age or adult PCD studies were retained as population-bound PCD evidence. Evidence from cystic fibrosis or bronchiectasis was treated as indirect. Protocols, conference abstracts, and preclinical studies were used only to describe ongoing research or future directions. Sources outside the scope of the review were excluded. No language restrictions were applied. J.Z. conducted the literature search and initial selection. L.C. reviewed the selected sources. Uncertainties were resolved through discussion.

## 2. A Clearance-Anchored Framework for Pediatric PCD Care

This review uses a clearance-anchored framework because impaired mucociliary transport is the initiating defect. The resulting secretion retention promotes chronic infection, inflammation, and progressive bronchiectasis [7,16]. ACT is therefore the pathophysiological anchor: it directly facilitates the movement and removal of retained secretions. Mucoactive treatment may facilitate this process but remains an adjunct rather than a separate pillar.

Pulmonary rehabilitation is a complementary functional pillar. It targets documented limitations in exercise capacity, respiratory muscle performance, and habitual activity [13,14]. It also supports participation, treatment confidence, and transition to self-management. The two pillars are mechanistically asymmetric and non-substitutable. Exercise may augment ventilation and cough-mediated secretion clearance [4,8]. However, rehabilitation cannot replace prescribed mechanical clearance. Conversely, technically effective clearance does not by itself reverse deconditioning or restore participation. Antimicrobial treatment remains essential when infection is present [4,5], but it operates beside rather than within either supportive-care pillar.

Clinical statements within this framework are interpreted using author-defined descriptive levels of evidence based on population relevance and study type. Level A evidence comprises direct pediatric PCD interventional or comparative studies. Level B evidence comprises pediatric PCD observational studies. Level C evidence comprises mixed-age, adult, or mixed-disease studies involving PCD. Level D evidence comprises indirect evidence from other respiratory diseases. Guideline or consensus recommendations (G/C), physiological rationale (PR), and the author-proposed framework (AP) are identified separately [4,5,7,8,17]. These levels summarize evidence provenance and directness for pediatric PCD; they do not constitute a formal assessment of certainty, risk of bias, or recommendation strength and should not be interpreted as GRADE or Oxford Centre for Evidence-Based Medicine levels. Accordingly, Level A evidence does not necessarily represent high-certainty evidence. The framework is compatible with the bronchiectasis treatable-traits concept linking mucus, infection, and inflammation but does not import its algorithm [18].

Five-step implementation workflow: To translate the framework into routine care, we propose five sequential steps. First, assess the current clinical problem, secretion burden, ACT performance, symptoms and exacerbation history, functional limitations, family support and preferences, and local resources. Second, set separate airway-clearance and functional goals. Third, select a developmentally appropriate ACT, teach and directly observe performance, and define child and caregiver roles. When a functional goal is identified, add age-appropriate physical activity, exercise training, or pulmonary rehabilitation. Fourth, choose feasible, goal-matched measures from the four monitoring domains rather than requiring every child to complete every assessment. Fifth, review clinical response, technique, adherence, burden, and feasibility after the initial monitored trial and at routine review. Reassess after exacerbations, meaningful symptom change, or developmental transition. Evidence for individual components is discussed in Section 3, Section 4 and Section 5.

**Illustrative clinical application:** Consider a hypothetical 10-year-old child with clinically stable PCD who has persistent wet cough and difficult expectoration despite prescribed ACT, which is often shortened because of school demands. Assessment identifies both a secretion-clearance target and a treatment-performance problem. Because the child can follow breathing instructions and prefers a portable device, OPEP is selected, taught, and directly observed [5,19]. At planned review, symptoms, expectoration, technique, adherence, and treatment burden are reassessed. Poor technique prompts re-teaching; adequate delivery without benefit prompts reconsideration of ACT choice, dose, or a selective adjunct, whereas improvement with acceptable burden supports continuation.

**Figure 1:**
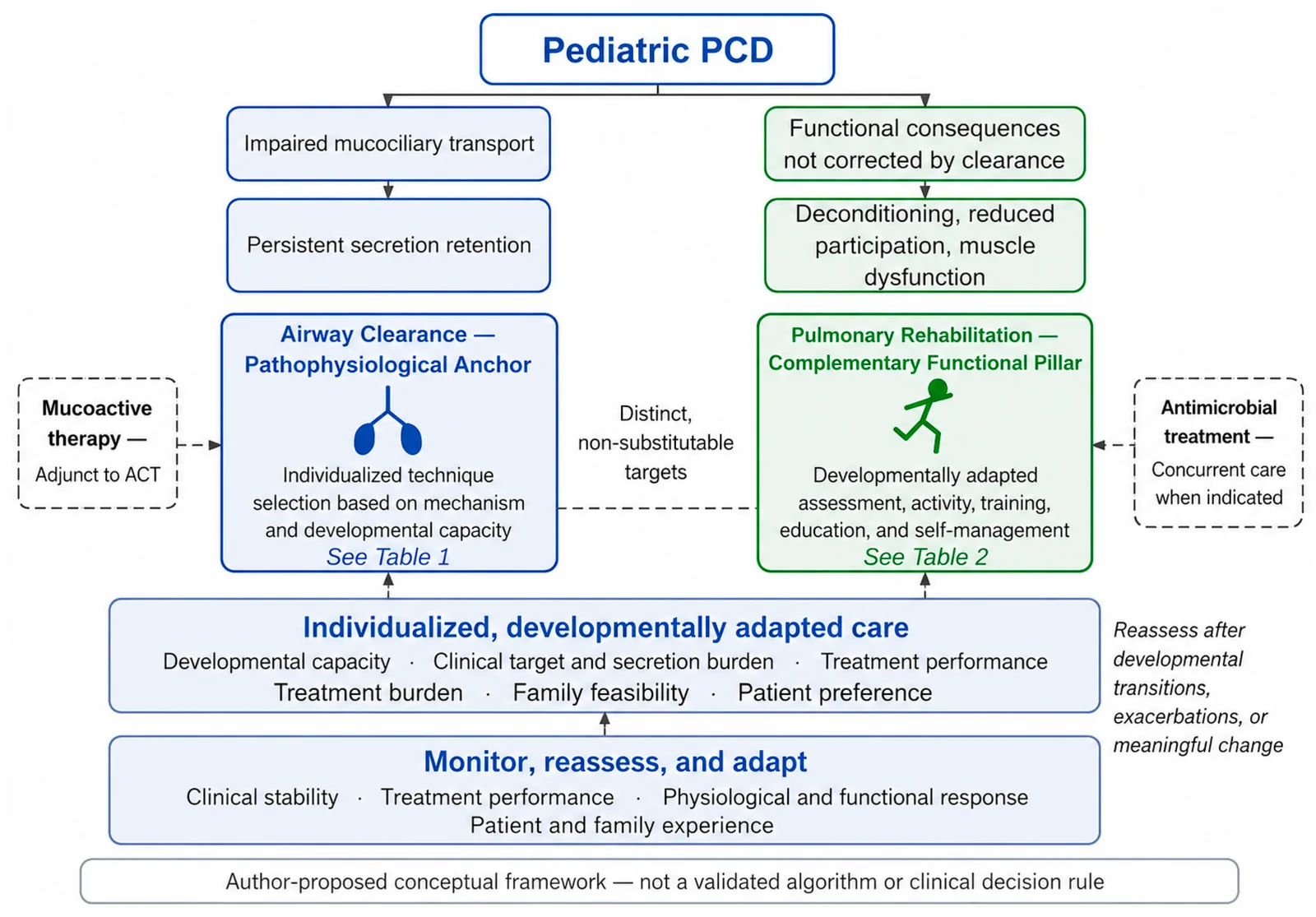
Author-proposed, clearance-anchored, dual-pillar framework for pediatric PCD care.

Figure 1 summarizes the two pillars, four-domain feedback, and reassessment after clinically meaningful change.

## 3. Airway Clearance in Pediatric PCD

ACT directly targets retained secretions, whereas antimicrobial and mucoactive therapies serve different roles. 

### 3.1. Mucoactive Therapy as an Adjunct

Mucoactive agents may alter airway hydration or mucus rheology but do not correct the ciliary defect [16,20]. A 2026 comparative study of clinically stable adolescents and adults with PCD found higher sputum elastic and viscous moduli and higher levels of several inflammatory markers than healthy controls [21]. These findings provide PCD-specific biological context but do not establish the efficacy of any mucoactive treatment. PCD expert consensus permits individualized use, particularly of hypertonic saline [5]. Pediatric observational data show that these therapies are commonly prescribed despite limited PCD-specific benefit–risk evidence [22]. Their clinical role is therefore adjunctive rather than routine. Use should be linked to a defined problem, such as tenacious secretions that impede ACT. Treatment should continue only when tolerability and prespecified clinical or patient-reported goals justify the added burden.

Among conventional mucoactive agents, hypertonic saline is the only one tested in a completed randomized trial designed specifically for PCD. In a double-blind crossover study, 22 adults aged 22–73 years received 7% hypertonic saline or 0.9% isotonic saline twice daily for 12 weeks, separated by a four-week washout [23]. The prespecified primary endpoint, St George’s Respiratory Questionnaire total score, did not improve significantly. A favorable signal occurred in the QoL-B Health Perception domain, a secondary outcome, and adverse events were more frequent but mild [23]. This adult study does not establish pediatric efficacy or effects on exacerbations or structural disease. It supports only a monitored trial in selected patients. Monitoring should cover bronchospasm, throat irritation, timing relative to ACT, expectoration, perceived benefit, and treatment burden. A retrospective two-center study of 35 infants diagnosed with PCD near birth found that regular inhaled hypertonic saline use was associated with less frequent *Pseudomonas aeruginosa* isolation but more inpatient respiratory exacerbations. Because treatment exposure was concentrated at one center and the study was small and non-randomized, these findings do not establish benefit or harm and require prospective confirmation [24].

Tolerability strategies are supported only indirectly. In an open-label study of 137 adults with non-CF bronchiectasis, 45 were initially intolerant to 7% hypertonic saline; 31 subsequently tolerated saline with 0.1% hyaluronic acid and 26 completed four weeks [25]. This suggests a short-term adult bronchiectasis option, not pediatric PCD efficacy. PCD Foundation consensus recommends inhaled bronchodilators on a case-by-case basis rather than routinely [4]. In a retrospective study of 82 children with PCD, 86 of 474 bronchodilator-response tests (18.1%) were positive, and 34 children (41%) had at least one positive test; however, the study did not evaluate bronchodilator treatment efficacy [26]. Routine bronchodilator pretreatment solely to augment clearance is therefore not supported. When bronchodilation is independently indicated for clinically relevant reversible airflow obstruction or when inhaled saline provokes bronchospasm, it can be coordinated before mucoactive therapy and ACT.

Routine recombinant human deoxyribonuclease I (rhDNase) is not supported. A 24-week trial in 349 adults with idiopathic bronchiectasis found more exacerbations and greater FEV_1_ decline with rhDNase than placebo [27], an indirect harm signal whose applicability to PCD is uncertain. An uncontrolled 2026 pilot involving eight adults with PCD-related bronchiectasis reported stable spirometry and a reduction in mean exacerbations from 3.1 to 2.3 over six months [28]. However, variable dosing, concurrent therapies, and the absence of a control group preclude efficacy inference. Any exceptional use should be specialist-supervised, goal-based, and subject to stopping criteria.

Evidence for inhaled mannitol remains indirect. In a 12-week double-blind trial of 343 participants aged 15–80 years with non-CF bronchiectasis, mannitol produced a modest difference in sputum weight but no clear clinically meaningful improvement in overall quality of life [29]. Mannitol should therefore remain an individualized extrapolation rather than a standard therapy in pediatric PCD. Oral N-acetylcysteine has limited direct PCD evidence. In a small double-blind, placebo-controlled crossover trial, 13 participants with PCD received oral NAC or placebo for two 3-month periods, with no improvement in clinical status or lung function [30]. An open-label randomized trial in adults with non-CF bronchiectasis reported fewer exacerbations, lower sputum volume, and improved quality of life with NAC, whereas lung function did not improve [31]; however, these findings cannot establish efficacy in PCD. Routine oral NAC is therefore not supported in pediatric PCD.

**Problem-oriented selection:** Mucoactive therapy should be considered only after ACT performance and the unresolved clearance problem have been reassessed. When tenacious secretions or difficult expectoration continue to limit effective clearance despite adequately performed ACT, and increasing airway hydration is a plausible target, an osmotic adjunct may be considered as a monitored therapeutic trial. Hypertonic saline has the most directly relevant PCD trial evidence for this purpose, although the evidence is limited, adult, and does not establish pediatric efficacy [5,23]. Mannitol remains an indirect, individualized extrapolation [29]. Routine oral NAC is not supported by the available direct PCD trial [30], and routine rhDNase is not supported given the limited PCD data and the indirect harm signal from non-CF bronchiectasis [27,28]. No validated PCD-specific rule currently matches a secretion phenotype to a particular mucoactive agent; choice and continuation should depend on the clinical target, tolerability, treatment burden, and prespecified clinical or patient-reported benefit.

### 3.2. Comparative Evidence for Airway Clearance Techniques

Comparative evidence does not establish a preferred ACT. The 2024 systematic review included two randomized crossover trials with 54 participants aged 6–20 years. It judged all outcomes to be of very low certainty and found no clear FEV_1_ or FVC advantage of device-supported techniques over conventional chest physiotherapy [12]. Absence of a detected difference is not proof of equivalence because the trials were small, single-center, and clinically heterogeneous.

Bingol and colleagues randomized 30 clinically stable participants to OPEP or conventional chest physiotherapy in a crossover design, with each technique performed for three months [32]. Changes in spirometry, oxygen saturation, and pulmonary exacerbation frequency did not differ significantly, while comfort favored OPEP. The study therefore supports OPEP as an acceptable option for some children and adolescents, not as a superior or disease-modifying technique. Gokdemir and colleagues compared high-frequency chest wall oscillation (HFCWO) with conventional chest physiotherapy in 24 clinically stable participants, again using a randomized crossover design [33]. Each intervention lasted five days; spirometry and oxygen saturation changed similarly, while comfort favored HFCWO. The short intervention periods restrict interpretation to immediate physiological responses, oxygenation, and patient comfort, rather than sustained effectiveness.

A later randomized feasibility study compared 12 weeks of home-based active cycle of breathing techniques with OPEP in 32 children and adolescents aged 6–18 years in Palestine [34]. Post-intervention FEV_1_, mid-expiratory flow, and six-minute walk distance favored OPEP, whereas FVC and FEV_1_/FVC did not differ significantly. Because feasibility was the primary purpose and the study was small and single-center, these efficacy signals are hypothesis-generating.

Together, the studies show that breathing-based and device-supported approaches can be delivered to clinically stable school-age children and adolescents. They provide little evidence regarding infants, preschool children, exacerbations, adherence, structural progression, or long-term outcomes. No ACT should therefore be presented as universally superior. A mechanism-based classification can guide initial choice without implying a hierarchy [35]. Selection should depend on the child’s ability to perform the technique correctly and consistently. Table 1 summarizes the key comparative ACT studies and their main limitations.

### 3.3. Selecting and Implementing an ACT

Because comparative evidence does not establish a device hierarchy [12], ACT selection should be individualized and revisited over time [5,19]. PCD-specific qualitative research indicates that physiotherapists consider age, disease severity, pulmonary and non-pulmonary factors, lifestyle, engagement, burden, and preference when adapting technique and dose [19]. A broader scoping review similarly grouped personalization factors into physical and psychosocial characteristics, ACT procedures, dose, response, and provider factors [36]. These sources describe practice rather than validate a matching rule.

Consensus-derived recommendations support an individualized routine with regular specialist review [5]. Pediatric bronchiectasis guidance provides indirect, guideline-derived support for developmentally appropriate progression from caregiver-assisted treatment toward active techniques and self-management [17,37]. Physiological rationale should connect the selected method to the immediate problem. The child must be able to generate or receive the intended airflow or oscillation. Mobilized secretions must then be removed by an effective huff, cough, or expectoration.

After the framework-level assessment and goal setting described in Section 2, chronological age should inform but not determine ACT choice. The prescription should specify frequency, duration, sequence with inhaled therapy, and the huffing or coughing component. It should also define device settings where relevant, cleaning, caregiver and child responsibilities, and an exacerbation plan [4,5]. Patient preference matters because acceptability can support sustained delivery, but preference alone does not establish physiological effectiveness. Secretion burden should be characterized by symptoms, expectoration, auscultatory or imaging context where clinically available, and change from baseline rather than by a single sputum-volume threshold. Family feasibility includes time, equipment access, cleaning capacity, travel, and the number of other daily treatments.

Bronchodilators and inhaled corticosteroids should be positioned separately from ACT. A positive bronchodilator response may occur in pediatric PCD without diagnosed asthma and does not by itself establish bronchodilator treatment efficacy [26]. PCD Foundation consensus permits bronchodilators on a case-by-case basis and does not recommend routine ICS [4]. In a cross-sectional study of 99 children with PCD, reversible airflow obstruction was not associated with atopy, elevated FeNO, or ICS prescribing, and objective evidence of airway eosinophilia was uncommon [38]. A 2026 PCD consensus further notes that bronchial hyperresponsiveness in PCD may often reflect a T2-low phenotype and that prospective studies of ICS efficacy in PCD are lacking [39]. ICS should therefore not be initiated solely because bronchodilator responsiveness is present; a separate clinical indication consistent with corticosteroid-responsive airway disease should be established. When bronchodilator therapy is independently indicated, its timing can be coordinated with inhaled therapy and ACT. A small physiological study of 12 children with PCD found a greater PEFR response to exercise than to inhaled salbutamol, suggesting that exercise before ACT may provide a useful non-pharmacological bronchodilator strategy when feasible; individual responses should be assessed, and effects on mucus-clearance outcomes remain unestablished [40]. Neither bronchodilators nor ICS should be added solely to enhance mucus clearance.

The first ACT prescription should be treated as a monitored therapeutic trial. Technique review should assess breathing pattern, resistance or settings, cough effectiveness, tolerance, time demands, and integration with school and family routines [5,19]. In a small genotype-defined in vivo study, mucociliary clearance was impaired in both DNAH5- and RSPH1-related PCD. Cough clearance was greater in the RSPH1 group than in the DNAH5 group [41]. This finding supports direct assessment of cough-mediated clearance rather than an assumption that it is uniform across PCD. PCD-specific trials of adherence interventions were not identified. A CF Cochrane review of two small randomized studies could not establish reliable effects on adherence or clinical outcomes [42]. Shared decision-making, re-teaching, written instructions, reminders, and simplification are therefore implementation supports rather than proven outcome-modifying interventions.

Missed or shortened sessions should prompt a non-judgmental review of knowledge, discomfort, competing responsibilities, equipment, fatigue, and perceived benefit. Indirect pediatric bronchiectasis evidence documents substantial caregiver burden and gaps in exacerbation knowledge [43]. When performance is inadequate, re-teaching or redesign should precede escalation to a more complex device. Persistent problems despite acceptable delivery instead justify reconsideration of mechanism, dose, adjunctive mucoactive therapy, or specialist support. A preference-sensitive choice is most defensible when two options have plausible mechanisms, can be performed adequately, and have no evidence-based superiority.

### 3.4. Escalation During Pulmonary Exacerbations

Exacerbation recognition should be based on sustained change from the individual baseline because wet cough and sputum may persist when a child is clinically stable. For research, expert consensus requires at least three of seven features. Five concern increased cough, changed sputum volume or color, increased breathlessness, a decision to start or change antibiotics for pulmonary symptoms, or malaise, fatigue, or lethargy. The remaining features are new or increased hemoptysis and temperature above 38 °C [44]. This standardizes trial reporting but is not a validated sole trigger for clinical treatment.

PCD consensus supports daily clearance, while pediatric bronchiectasis guidance indirectly supports increasing ACT frequency during exacerbations [4,17]; neither provides an evidence-based escalation dose. An international cohort of 660 people with PCD recorded 1026 self-reported exacerbations, estimated at 3.1 events per person-year, but did not test a clearance regimen [45]. Escalation may involve additional sessions or shorter, more frequent sessions, direct technique review, increased caregiver or physiotherapist assistance, more frequent huffing or coughing, and a previously tolerated mucoactive adjunct. The plan should remain achievable during fatigue or breathlessness and should be modified if safe, effective completion is not possible.

ACT intensification complements rather than replaces antimicrobial therapy. PCD consensus recommends using previous respiratory cultures to guide antibiotic choice [4]. Pediatric bronchiectasis guidance provides indirect recommendations on clinical severity, response to oral treatment, escalation to intravenous therapy, and a commonly used 14-day antibiotic course [17]. These should not be described as PCD trial results. Failure to improve should trigger reassessment of microbiology, antibiotic delivery, retained secretions, technique, complications, and the need for hospital or specialist support.

The previous maintenance plan should not be resumed automatically without first assessing recovery. After a first exacerbation treated with intravenous antibiotics, 7 of 30 children (23%) did not regain more than 90% of baseline FEV_1_ within three months [46]. In a 2025 single-center cohort of 85 children with 337 exacerbations, 297 (88%) were treated with oral antibiotics. FEV_1_ exceeded 90% of stable baseline in 73.2% at approximately three months and 84.2% within 12 months. Younger age and non-response after the preceding exacerbation were associated with subsequent non-response [47]. These observational data do not isolate the effect of ACT escalation. They support post-exacerbation review of symptoms, secretion burden, treatment performance, lung function, and functional status.

## 4. Pediatric Pulmonary Rehabilitation

Pulmonary rehabilitation is used here as a developmentally adapted package of functional assessment, habitual physical activity, structured training when indicated, education, behavioral support, and longitudinal reassessment. It complements ACT but does not replace mechanical clearance.

### 4.1. Clinical Rationale and PCD-Specific Evidence

The clinical rationale is functional rather than mucociliary. Among 44 clinically stable participants aged 6–29 years, peak oxygen uptake was lower than in controls and abnormal in 34% [14]. In another cross-sectional study, 27 patients had lower inspiratory muscle performance, six-minute walk distance, energy expenditure, and selected quality-of-life scores than 28 controls [13]. These observations identify potentially treatable limitations but do not show that a standardized rehabilitation program reverses them or changes long-term respiratory disease.

The strongest direct pediatric intervention evidence is a single-center randomized trial of active video gaming. Thirty-six clinically stable participants aged 6–18 years were randomized. Thirty-two completed the study and were analyzed. Participants received eight weeks of moderate-intensity gaming plus usual ACT or ACT alone. Sessions lasted 40 min three times weekly, and six-minute walk distance was the primary outcome [15]. Between-group changes favored gaming for several short-term functional and QOL-PCD outcomes. With 16 participants per group, multiple outcomes, and no long-term follow-up, the study supports the feasibility and short-term effect of one gamified modality. It does not establish a standardized rehabilitation program or durable disease modification.

A related analysis of an overlapping registered study cohort found lower step-test performance, cough strength, and thoracoabdominal mobility in the PCD group. Cough strength correlated with expiratory muscle strength and exercise performance [48]. A separate study compared 15 children with Kartagener syndrome, 23 with PCD without Kartagener syndrome, and 27 controls. It found group differences in lung function, respiratory muscle strength, six-minute walk distance, and daily steps; the pattern differed between PCD subgroups [49]. These data demonstrate heterogeneity but neither establish phenotype-specific treatment effects nor justify different rehabilitation protocols based on laterality status.

Additional cross-sectional evidence in 20 patients aged 8–18 years and 20 matched controls found less favorable respiratory muscle strength, incremental shuttle walk performance, multidimensional fitness, and activities-of-daily-living performance in PCD [50]. A comparison of 23 children with PCD and 23 healthy peers associated six-minute walk and incremental shuttle performance with peak oxygen uptake. CPET additionally identified ventilatory inefficiency and chronotropic abnormalities not fully characterized by field tests [51]. Test selection should follow the clinical question. Field tests can support practical repeated measurement, whereas CPET is reserved for detailed physiological assessment with appropriate expertise and reference values [52,53].

Two related reports from an overlapping registered cross-sectional cohort further characterized the functional phenotype of pediatric PCD. Deltoid strength was lower despite preserved upper-extremity functional exercise performance. Dynamic balance, six-minute walk distance, and lower-extremity strength were also lower. During walking, the decrease in quadriceps muscle oxygen saturation was greater than in healthy peers [54,55]. These findings support targeted assessment when clinically indicated but do not establish treatment effects.

Move-PCD is a seven-center German randomized superiority trial protocol aiming to enroll 158 participants aged 7–55 years and compare a six-month individualized, supported physical-activity program with usual activity advice [56]. The primary endpoint is change in the QOL-PCD physical-functioning domain at six months, with physical activity, motor performance, and lung function among secondary outcomes. Because the cited publication is a protocol, it informs intervention design and future evidence but provides no efficacy results. Indirect CF evidence from ACTIVATE-CF likewise illustrates that increased vigorous activity and exercise capacity need not translate into improved spirometry [57]. Because Move-PCD includes participants aged 7–55 years, any eventual overall result will require age-stratified interpretation before being applied to children. Table 2 summarizes the key completed intervention studies informing exercise and pulmonary rehabilitation in pediatric PCD care.

Completed randomized rehabilitation evidence in pediatric PCD is limited to one small, single-center, 8-week trial involving participants aged 6–18 years [15]. No completed randomized rehabilitation trial in infants or preschool-aged children was identified in the literature reviewed.

### 4.2. Developmentally Adapted Rehabilitation

Developmental adaptation should follow competence and readiness rather than rigid age thresholds. Across childhood, rehabilitation should progress from caregiver-supported movement and play, through enjoyable child-selected activity, toward increasingly structured aerobic and strength-based training, symptom monitoring, goal setting, and adolescent self-management. Prescription should reflect motor competence, cognition, clinical stability, functional limitation, access, family context, previous treatment experience, and patient preference. Pragmatic developmental considerations for ACT and pulmonary rehabilitation are summarized in Table 3.

For younger children, the priority is to protect normal movement and participation without conflating play, walking, or breathing games with prescribed ACTs. For school-age children, activity may become more structured, but modality, frequency, duration, and intensity should be individualized rather than presented as a validated PCD-specific dose. The active-video-gaming trial supports one short-term option, not a general rehabilitation prescription [15]. Assessment should be used when feasible and clinically informative.

During adolescence, planning and monitoring should progressively transfer to the young person. Care should address barriers related to fatigue, embarrassment, privacy, access, and school or work demands. Reduced participation should be explored rather than labeled simply as non-adherence. Transition planning should begin before adult transfer. It should include a written care summary and progressive responsibility, supported by PCD expert consensus and indirect transition guidance [5,62].

**Table 3:** Developmental considerations for airway clearance and pulmonary rehabilitation in pediatric PCD.

Developmental Stage (Pragmatic Age Guide)	ACT Considerations	Rehabilitation Priorities and Common Strategies	Child–Caregiver Role
**Infants and toddlers** (birth to <3 years)	Caregiver assistance is generally needed; positioning, manual techniques, or assisted approaches may be selected according to secretion burden, tolerance, and specialist assessment. Independent self-administered ACT is generally not feasible at this stage [5,17,37].	Protect normal movement, play, and motor development; avoid unnecessary activity restriction. No validated PCD-specific rehabilitation program or exercise dose is available for this age group.	**Caregiver-led.** Integrate treatment with sustainable feeding, sleep, and family routines.
**Preschool children** (3 to <6 years)	Caregiver support remains important. Simple active components or selected PEP-based approaches may be introduced when cooperation and technique permit; chronological age alone should not determine progression [17,37].	Encourage enjoyable play-based physical activity, movement competence, and participation. Play or breathing games should not be presented as substitutes for prescribed ACT.	**Caregiver-guided,** with increasing child choice and participation.
**School-age children** (6–11 years)	Progress toward active or partly self-administered techniques when the child can perform them correctly, including huff/FET, ACBT, PEP, or OPEP as appropriate. Comparative PCD studies support feasibility but do not establish a universally superior technique [32,33,34]; PCD-specific qualitative evidence supports individualized selection [19].	Encourage individualized aerobic and strength-based activity, sport, or other enjoyable exercise. Active video gaming is one PCD-specific short-term intervention studied in children aged 6–18 years, but no universal rehabilitation dose is established [15].	**Shared responsibility.** Involve the child in goal-setting, technique, and monitoring while caregivers support consistency and logistics.
**Adolescents** (12–18 years)	Increase independent ACT and self-monitoring where technically appropriate. Reassess technique and dose in relation to symptoms, treatment burden, preferences, school/work demands, and transition readiness [5,19,63].	Progress toward individualized aerobic and strength-based activity guided by documented functional limitations [5,13,48]; consider targeted IMT when reproducible inspiratory weakness is present [58]. Address participation, fatigue, self-management, and preparation for adult care [5,62].	**Progressive autonomy.** Transfer planning and treatment responsibility toward the adolescent while retaining family support as needed [5,62,63].

**Note:** Age ranges are pragmatic developmental guides rather than validated treatment thresholds. Developmental capacity and cooperation should guide decisions alongside the clinical target, secretion burden, treatment performance and burden, family feasibility, and patient preference; chronological age should inform, but not determine, selection. ACT guidance for younger children is largely consensus- or guideline-derived and indirect, whereas direct comparative PCD evidence is concentrated in school-age children and adolescents [32,33,34]. Direct PCD rehabilitation intervention evidence is limited, and no completed rehabilitation trial in infants or preschool-aged children was identified by the 24 June 2026 search cut-off.

### 4.3. Targeted and Remote Interventions

Targeted inspiratory muscle training (IMT) should be linked to a reproducible impairment rather than prescribed routinely. In a single-center mixed-age pilot, 19 participants with PCD and eight with CF completed one month of home-based IMT. Maximal inspiratory pressure improved in the PCD subgroup, whereas spirometry, maximal expiratory pressure, and lung clearance index did not change significantly [58]. The uncontrolled design, broad age distribution, short duration, and absence of exercise and quality-of-life outcomes establish feasibility only.

Supporting evidence is indirect. In a pediatric CF randomized trial, adding IMT to comprehensive chest physiotherapy produced greater improvement in inspiratory pressure but no additional between-group benefit for spirometry, postural stability, or six-minute walk distance [60]. In pediatric bronchiectasis, 17 children entered a single-group respiratory muscle training study and 15 completed 24 weeks. Pre–post improvements were reported in respiratory pressures, several spirometric measures, cough-related quality of life, and fatigue [61]. However, causal inference is limited without a control group. IMT may therefore be considered only for cooperative children with reproducible inspiratory weakness or another defined functional indication. Treatment should have prespecified goals, technique review, and stopping criteria.

Remote or hybrid support may reduce travel and scheduling barriers, but no PCD-specific efficacy trial was identified. A pediatric CF randomized trial involved children aged 6–13 years. At 12 weeks, 6MWD and selected child anxiety and depression scores favored telerehabilitation. FEV_1_, most quality-of-life outcomes, and caregiver anxiety and depression showed no clear between-group benefit [59]. A pediatric CF scoping review identified five heterogeneous studies involving exercise, treatment-management support, or symptom monitoring [64]. This indirect evidence supports feasibility, not equivalence to in-person care or effectiveness in pediatric PCD.

Remote review may support activity coaching, symptom diaries, transition care, and selected observation of ACT performance. Periodic in-person assessment may still be necessary for equipment fitting, detailed functional testing, and technique correction. Delivery should be selected according to clinical need, digital access, privacy, supervision, and treatment burden, with predefined goals and review criteria. Hybrid follow-up may support transition when responsibilities, escalation contacts, and in-person requirements are clearly documented.

## 5. Monitoring Treatment Response

No validated pediatric PCD composite tool integrates clinical stability, treatment performance, physiological and functional response, and patient and family experience. We therefore propose four linked monitoring domains. The model is author-proposed and intended to structure decisions, not to serve as a validated score or decision rule.

### 5.1. Clinical Stability

Clinical stability should be judged against the child’s usual baseline because wet cough and upper- and lower-airway symptoms may persist between exacerbations [2,5]. Records should capture sustained changes in cough, sputum volume or color, breathlessness, fatigue, sleep, appetite, activity tolerance, school participation, antibiotic courses, unscheduled care, admissions, and time to recovery. The PCD trial definition can standardize event documentation but is not a sole clinical treatment threshold [44].

Recovery may be delayed or incomplete after pulmonary exacerbations, as summarized in Section 3.4 [47]. Such trajectories should prompt reassessment of infection treatment, secretion burden, ACT delivery, inhaled adjuncts, and functional status.

### 5.2. Treatment Performance

Treatment performance asks what was actually delivered. For ACT, assessment should include completed frequency and duration, sequence with inhaled treatment, breathing pattern, huffing or coughing, device resistance or settings, cleaning, caregiver assistance, and reasons for missed or shortened sessions. For rehabilitation, it should include modality, completed dose, achieved intensity where measurable, progression, supervision, symptoms, recovery, and barriers. A prescription or device-use statement alone does not establish treatment quality.

Consensus and PCD-specific qualitative evidence support direct observation because technique, family roles, and feasibility change with development [5,19]. Digital diaries, wearable or device-recorded data, and video review may supplement assessment, but no PCD-specific trial has shown that these tools improve outcomes. Within this framework, inadequate implementation is corrected through re-teaching, simplification, or redesign before physiological non-response is declared or a more complex intervention is introduced.

### 5.3. Physiological and Functional Response

Physiological monitoring should not rely on spirometry alone. Structural disease and ventilation inhomogeneity may not be fully captured by FEV_1_ [65,66]. In a prospective multicenter pediatric cohort, longitudinal CT scoring showed increasing structural airway disease with age, driven by progressive bronchiectasis and greater odds of mucus plugging [67]. Among 17 children aged 3–12 years completing acceptable multiple-breath washout (MBW) and spirometry, lung clearance index (LCI) was abnormal in 10 (59%) and FEV_1_ in three (18%) [68]. A systematic review found LCI generally more sensitive than spirometry but methodologically heterogeneous [69]. Pediatric LCI repeatability was high on the same day and moderate at 28 days, with intraclass correlation coefficients of 0.95 and 0.71 [70]. In a multicenter cohort of 90 individuals aged 3–41 years, higher LCI was also associated with a greater risk of subsequent pulmonary exacerbation, supporting potential prognostic value at the visit level [71]. Repeatability does not establish responsiveness to treatment or a clinically important change threshold.

PROVALF-PCD found that an intra-individual relative change in FEV_1_ exceeding 25% between visits without a clinician-defined pulmonary exacerbation may exceed expected test variability and therefore represent a physiologically relevant change [72]. However, this value should not be used as an individual clinical threshold, because smaller changes may still be clinically meaningful.

Functional outcomes should match the rehabilitation goal. Field tests can provide practical repeated measures, whereas CPET offers more detailed physiological assessment when appropriate expertise and reference equations are available [52,53]. As discussed in Section 4.1, CPET may identify abnormalities not fully characterized by field tests [51]. Respiratory muscle strength, cough strength, mobility, fitness, daily activity, and ADL measures may identify other limitations [13,48,50].

### 5.4. Patient and Family Experience

Patient- and family-reported assessment should cover symptoms, physical functioning, emotional and social effects, perceived benefit, treatment burden, and satisfaction. QOL-PCD has validated child (6–12 years), adolescent (13–17 years), and parent-proxy versions for children aged 6–12 years [73]. The same age-appropriate version and reporter should be used longitudinally when possible. Validation supports measurement, but no pediatric minimal clinically important difference was identified for routine treatment decisions (Table 4).

Burden should be evaluated separately from respiratory symptoms. Qualitative PCD research describes the daily effects of physiotherapy and gradual transfer of responsibility from family to child [63]. A comparative study of children aged 6–13 years documented caregiver burden in PCD, although mean Zarit scores were lower than in the CF group [74]. Relevant questions include time demands, family conflict, school disruption, embarrassment, privacy, fatigue, caregiver confidence, and transition readiness.

The domains should be interpreted together. Acceptability may support sustained treatment but does not prove physiological effectiveness. Stable spirometry does not justify an impractical regimen. Adequate delivery with deterioration prompts reassessment of infection, secretion burden, ACT, adjuncts, and rehabilitation. Functional improvement without secretion-related improvement may indicate rehabilitation benefit while clearance remains insufficient. The four-domain model therefore makes discordance visible, but its combinations and action thresholds require prospective validation. Decisions should identify the domain driving the change. They should also specify the outcome expected at the next review, making later reassessment more interpretable.

## 6. Evidence Gaps and Future Directions

Future research should prioritize multicenter comparative trials of ACTs because current PCD evidence is based on small, short, and very low-certainty comparative studies [12]. Rehabilitation strategies also require multicenter evaluation, as completed intervention evidence is limited to one small single-center randomized trial and one uncontrolled pilot [15,58]. Trials should define intervention components, dose, and fidelity and match study design to the intended outcome. They should report pediatric subgroups, adverse events, and treatment contamination. They should also determine whether rehabilitation adds functional benefit when ACT is held stable. The Move-PCD protocol provides a useful model for a supported physical-activity intervention but does not yet provide efficacy evidence [56]. Harmonized outcomes are also needed. The BEAT-PCD core outcome set specifies core outcomes for pulmonary intervention trials [75]. ACT and rehabilitation studies should additionally consider intervention-specific measures of treatment performance, secretion burden, treatment burden, exercise capacity, muscle performance, activity, participation, and patient-defined goals. Responsiveness and clinically interpretable thresholds must be established before the proposed four-domain monitoring framework can direct care.

Implementation research should compare digital, hybrid, and in-person models while assessing reach, adoption, fidelity, maintenance, cost, workforce requirements, family burden, and equity. An Italian descriptive survey of 29 physiotherapy units involved in pediatric PCD and non-CF bronchiectasis care documented heterogeneity in functional testing, exercise-program delivery, and device availability. It also reported involvement in telemedicine but did not assess patient-level outcomes [76]. A Swiss cross-sectional survey found that professional respiratory physiotherapy and exercise recommendations were not consistently implemented in routine PCD care [77]. This finding underscores the need to evaluate multidisciplinary implementation rather than prescription alone. Remote ACT review, exercise support, diaries, wearables, and transition care therefore require direct evaluation, together with low-cost alternatives and clear indications for in-person assessment.

Resource-adapted implementation should preserve technical quality and longitudinal review while matching care to local capacity (AP). A basic package may combine initially taught and periodically reviewed breathing and cough techniques with individually selected caregiver-assisted or equipment-free ACTs [5,17,19]. Physical activity may use walking, play, stairs, simple resistance exercise, or a feasible home plan. Follow-up can use symptom change, exacerbations, direct observation of ACT, simple activity records, and, where available, spirometry and the 6MWT. LCI, CPET, oscillatory devices, intensive specialist physiotherapy, and multidisciplinary rehabilitation should be problem-oriented additions, not mandatory at every visit. Their absence does not preclude meaningful longitudinal monitoring. However, low-cost approaches should not be assumed equivalent to specialist equipment or formal rehabilitation [8,12]. Infection, deterioration, poor response, or technical difficulty should prompt reassessment and referral where feasible.

Mechanism-directed therapies should be evaluated not only for short-term biological or spirometric effects but also for their impact on secretion burden, function, total treatment burden, and the continuing need for ACT or rehabilitation. CLEAN-PCD reported a 1.5-percentage-point greater change in percent-predicted FEV_1_ after 28 days of idrevloride in hypertonic saline than with hypertonic saline alone, but long-term outcomes and safe ACT reduction remain unknown [78]. *DNAI1* mRNA studies [79], the RCT1100 phase 1 program [80], and *Dnah9*-mutant models [81] represent preclinical or early-stage development rather than established clinical efficacy. Future trials should prespecify concurrent supportive care and prospective criteria for treatment de-escalation, safety monitoring, and reinstatement.

## 7. Conclusions

Impaired mucociliary transport makes airway clearance the pathophysiological anchor of pediatric PCD care. Pulmonary rehabilitation is a complementary functional pillar addressing exercise capacity, muscle performance, physical activity, participation, and progressive self-management. It cannot replace mechanical secretion clearance, and effective clearance alone does not resolve deconditioning. Mucoactive therapy remains a problem-oriented, monitored adjunct rather than a separate pillar. No ACT has been shown to be universally superior [12]. Technique and dose should therefore be individualized, directly observed, and reassessed longitudinally [5,19]. Within this framework, decisions consider developmental capacity, the clinical target, secretion burden, treatment performance and burden, family feasibility, and patient preference. Rehabilitation should progress from caregiver-supported activity and play toward structured conditioning, targeted training where indicated, adolescent autonomy, and transition planning. The review’s distinctive contribution is an author-proposed pediatric framework integrating non-substitutable pillars, developmental stage, evidence provenance, and four-domain reassessment. It is not a validated algorithm, consensus pathway, or clinical decision rule.

## Figures and Tables

**Table 1:** Key comparative studies of airway clearance techniques in primary ciliary dyskinesia.

Study and Evidence Source	Design, Participants, and Intervention	Key Outcomes and Findings	Main Limitations
**Bingol et al.** [32]—Mixed-age PCD evidence (Level C)	Randomized crossover; *n* = 30, ages 6–20 years; OPEP versus conventional chest physiotherapy, twice daily for 3 months each, with a 15-day washout.	Spirometry and exacerbation outcomes showed no between-method difference. OPEP was rated more comfortable; no adverse effects were reported.	Small, single-center, mixed-age study; short treatment periods and no structural, quality-of-life, or long-term outcomes.
**Gokdemir et al.** [33]—Direct pediatric PCD evidence (Level A)	Randomized crossover; *n* = 24, ages 7–18 years; HFCWO versus conventional pulmonary rehabilitation, each for 5 days with a 2-day washout.	Selected pulmonary-function outcomes improved similarly; no between-method difference. Comfort favored HFCWO.	Small, single-center, very short study; different treatment settings and no exacerbation, quality-of-life, or long-term outcomes.
**Fashho et al.** [34]—Direct pediatric PCD evidence (Level A)	Randomized parallel feasibility study; *n* = 32, ages 6–18 years; home-based ACBT versus OPEP for 12 weeks.	FEV_1_, MEF_25–75_, and 6MWT favored OPEP; FVC and FEV_1_/FVC did not differ. Findings were hypothesis-generating and did not establish universal superiority of OPEP.	Small, single-center, feasibility-first study; short follow-up and no exacerbation, structural, or long-term outcomes.

Abbreviations: ACBT, active cycle of breathing techniques; FEV_1_, forced expiratory volume in 1 s; FVC, forced vital capacity; HFCWO, high-frequency chest wall oscillation; MEF_25–75_, maximum expiratory flow between 25% and 75% of FVC; OPEP, oscillatory positive expiratory pressure; PCD, primary ciliary dyskinesia; 6MWT, six-minute walk test.

**Table 2:** Key completed intervention studies informing exercise and pulmonary rehabilitation in pediatric PCD care.

Study and Evidence Source	Design, Participants, and Intervention	Key Outcomes and Findings	Main Limitations
**Sonbahar-Ulu et al.** [15]—Direct pediatric PCD evidence (Level A)	Assessor-blinded randomized trial; 36 randomized, 32 completed and analyzed, ages 6–18 years; active video gaming plus usual ACT versus usual ACT, 40 min three times weekly for 8 weeks.	Gaming improved 6MWD and selected muscle, activity, and quality-of-life measures versus control, supporting a short-term effect of this modality.	Small, single-center study with multiple outcomes and no later follow-up; it does not establish a universal program or disease-modifying effect.
**Gur et al.** [58]—Mixed-age PCD/CF evidence (Level C)	Prospective uncontrolled pilot; 29 recruited, 27 analyzed (19 PCD, 8 CF), mean age 18.4 years; home IMT at 50% MIP twice daily for 1 month, with 1-month follow-up.	MIP improved and remained higher at follow-up, including in the PCD subgroup; spirometry, LCI, and MEP did not change.	Small, uncontrolled mixed-diagnosis and mixed-age sample with self-reported adherence; no exercise, quality-of-life, or exacerbation outcomes.
**ACTIVATE-CF.** [57]—Indirect cystic fibrosis evidence (Level D)	Multicenter randomized trial; *n* = 117 with CF, aged ≥ 12 years; three additional vigorous-activity hours weekly with support versus usual behavior for 12 months.	Activity and exercise capacity increased, but ppFEV_1_ favored control at 6 months; other outcomes showed no clear benefit.	Under-recruited, with control contamination and adherence challenges. Indirect evidence; PCD efficacy cannot be inferred.
**Kenis-Coskun et al.** [59]—Indirect pediatric cystic fibrosis evidence (Level D)	Single-blind randomized trial (outcome assessor blinded); 30 randomized, 28 completed and analyzed, ages 6–13 years with CF; supervised telerehabilitation comprising high-intensity interval and postural exercises three times weekly versus no added exercise for 12 weeks.	At 12 weeks, 6MWD and selected child anxiety/depression scores favored telerehabilitation; FEV_1_, most quality-of-life outcomes, and caregiver anxiety/depression showed no clear between-group benefit.	Small, short, single-center study; ACT was unmonitored. Indirect evidence; PCD efficacy cannot be inferred.
**Zeren et al.** [60]—Indirect pediatric cystic fibrosis evidence (Level D)	Randomized trial; 36 randomized, all 36 completed and were analyzed, ages 8–18 years with CF; chest physiotherapy plus IMT at 30% MIP versus physiotherapy alone for 8 weeks.	Both groups improved; only MIP showed an additional between-group gain with IMT.	Small, short, single-center study; no long-term clinical outcomes. Indirect evidence; PCD efficacy cannot be inferred.
**Chen et al.** [61]—Indirect pediatric bronchiectasis evidence (Level D)	Single-group pre/post-study; *n* = 17 with bronchiectasis, mean age 11.05 years; progressive inspiratory and expiratory threshold training five days weekly for 24 weeks; 15 completed.	Respiratory pressures, selected spirometric indices, cough-related quality of life, and fatigue improved from baseline.	Very small, uncontrolled study with device-use dropouts; causality is uncertain. Indirect evidence; PCD efficacy cannot be inferred.

The table includes two completed studies involving participants with PCD and four clearly identified indirect studies from other respiratory diseases. Move-PCD [56] is a published protocol, with no peer-reviewed efficacy results available by the 24 June 2026 search cut-off, and is therefore not listed as a completed intervention study. Abbreviations: ACT, airway clearance technique; CF, cystic fibrosis; FEV_1_, forced expiratory volume in 1 s; IMT, inspiratory muscle training; LCI, lung clearance index; MEP, maximal expiratory pressure; MIP, maximal inspiratory pressure; PCD, primary ciliary dyskinesia; ppFEV_1_, percent-predicted FEV_1_; 6MWD, six-minute walk distance.

**Table 4:** Major clinical recommendations and evidence sources in pediatric PCD care.

Major Recommendation or Practice Consideration	Main Evidence Basis and Source	Overall Interpretation
Regular, individualized ACT should remain a core component of pediatric PCD care.	PCD consensus [4,5] and pathophysiology [7,16]—G/C + PR.	Consensus- and physiology-based; long-term comparative trials are lacking.
No ACT has demonstrated universal superiority; selection should use a monitored therapeutic trial, direct observation, and reassessment.	PCD comparative studies [32,33,34] (two Level A studies and one Level C study) and qualitative personalization study [19].	Direct but limited evidence from small, short-term studies; no technique has established universal superiority. Personalization is supported by qualitative research.
During exacerbations, ACT may be intensified as an adjunct to antimicrobial treatment, followed by assessment of recovery.	PCD treatment consensus [4], exacerbation definition [44], and bronchiectasis guidance [17] (G/C), with pediatric PCD post-exacerbation recovery data [47] (Level B).	Consensus-led; the optimal dose and independent effect of intensified ACT remain untested.
Mucoactive treatments should be considered as problem-oriented, monitored adjuncts rather than prescribed routinely.	PCD mechanistic and treatment studies [21,23,28] (Level C), pediatric PCD observational evidence [24] (Level B), and mixed-disease PCD NAC trial evidence [30] (Level C), supplemented by indirect bronchiectasis trials [27,29,31] (Level D).	Direct PCD evidence remains limited and mixed; pediatric PCD efficacy and optimal use remain unestablished.
Bronchodilators and inhaled corticosteroids should be positioned separately from ACT. Bronchodilators should be considered case by case when independently indicated; ICS should not be used routinely or initiated solely on the basis of bronchodilator responsiveness.	PCD Foundation consensus [4] (G/C); pediatric PCD bronchodilator-response study [26] (Level B); pediatric PCD ICS study [38] (Level B); small pediatric PCD exercise–bronchodilator comparison [40] (Level A); and 2026 PCD ICS consensus [39] (G/C).	Bronchodilator responsiveness may occur in pediatric PCD but does not establish asthma, chronic bronchodilator treatment benefit, or ICS responsiveness. Prospective therapeutic evidence remains lacking.
Exercise and pulmonary rehabilitation complement rather than replace ACT and should be adapted to capacity and developmental readiness.	PCD gaming trial [15] (Level A), supported by a mixed-diagnosis study [58] (Level C) and indirect cystic fibrosis studies [57,59] (Level D).	Direct evidence is limited to active video gaming; broader rehabilitation evidence is mixed-diagnosis or indirect, and no universal program or dose is established.
Physiological and functional assessments should be selected according to the clinical question rather than relying on spirometry alone.	PCD physiological and cohort studies [67,68,69,71,72] (two Level B studies and three Level C studies).	PCD-specific observational evidence; measures are complementary, and clinically actionable treatment-response thresholds remain incompletely validated.
The author-proposed monitoring framework integrates clinical stability, treatment performance, physiological and functional response, and patient and family experience.	Evidence for individual components [5,37,48,63,66]; their integration is AP.	Author-proposed and unvalidated as an integrated framework.

Abbreviations: ACT, airway clearance technique; PCD, primary ciliary dyskinesia. The descriptive evidence levels summarize evidence source and directness for pediatric PCD: Level A, direct pediatric PCD interventional or comparative evidence; Level B, pediatric PCD observational evidence; Level C, mixed-age, adult, or mixed-disease evidence involving PCD; and Level D, indirect evidence from other respiratory diseases. G/C denotes guideline or consensus recommendations; PR, physiological rationale; and AP, author-proposed framework. These levels are not a formal assessment of evidence certainty, risk of bias, or recommendation strength, and Level A does not necessarily indicate high-certainty evidence.

## Data Availability

No new data were created or analyzed in this study. Data sharing is not applicable to this article.
